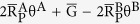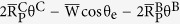# Wetting theory for small droplets on textured solid surfaces

**DOI:** 10.1038/srep37813

**Published:** 2016-11-29

**Authors:** Donggyu Kim, Nicola M. Pugno, Seunghwa Ryu

**Affiliations:** 1 Department of Mechanical Engineering, Korea Advanced Institute of Science and Technology (KAIST), 291 Daehak-ro, Yuseong-gu, Daejeon 34141, Republic of Korea; 2 Laboratory of Bio-Inspired and Graphene Nanomechanics, Department of Civil, Environmental, and Mechanical Engineering, University of Trento, Trento, Italy; 3 Center for Materials and Microsystems, Fondazione Bruno Kessler, Trento, Italy; 4 School of Engineering and Materials Science, Queen Mary University of London, Mile End Road, London, United Kingdom

## Abstract

Conventional wetting theories on rough surfaces with Wenzel, Cassie-Baxter, and Penetrate modes suggest the possibility of tuning the contact angle by adjusting the surface texture. Despite decades of intensive study, there are still many experimental results that are not well understood because conventional wetting theory, which assumes an infinite droplet size, has been used to explain measurements of finite-sized droplets. Here, we suggest a wetting theory applicable to a wide range of droplet size for the three wetting modes by analyzing the free energy landscape with many local minima originated from the finite size. We find that the conventional theory predicts the contact angle at the global minimum if the droplet size is about 40 times or larger than the characteristic scale of the surface roughness, regardless of wetting modes. Furthermore, we obtain the energy barrier of pinning which can induce the contact angle hysteresis as a function of geometric factors. We validate our theory against experimental results on an anisotropic rough surface. In addition, we discuss the wetting on non-uniformly rough surfaces. Our findings clarify the extent to which the conventional wetting theory is valid and expand the physical understanding of wetting phenomena of small liquid drops on rough surfaces.

The contact angle is a material property determined by the surface tensions between substrate, liquid, and vapor^1^. Because the materials with extremely small or large contact angles, i.e., with super hydrophilicity or super hydrophobicity, are applicable in many ways, there have been a myriad of studies that have investigated tuning the contact angle via the surface roughness of the substrate based on the conventional wetting theory of rough surface with Wenzel (W), Cassie-Baxter (CB), and Penetrate (P, which is also referred to as hemi-wicking^2^) modes^3^,^4^,^5^,^6^,^7^,^8^,^9^.

However, the conventional wetting theory^1^,^10^,^11^,^12^ assumes that the liquid droplet is much larger than the characteristic scale of the surface roughness, which frequently is not justifiable in many experiments. Therefore, the contact angle predictions using the conventional theory differ from experimental results when the droplet size is small^13^,^14^,^15^,^16^,^17^,^18^,^19^. The conventional theory considers a straight boundary between the liquid and the vapor regardless of the three-phase contact line location or the droplet size^1^,^10^,^11^,^12^,^20^. However, because the realistic contour of a liquid droplet forms part of a sphere^21^, the assumption does not hold when the droplet size to surface texture scale ratio is small. There have been several studies to overcome the limitations of the conventional wetting theories for the small droplet, based on wetting free energy calculations. As a pioneer of the subject, Marmur *et al*.^12^,^13^ modelled the 2D circular droplet on the heterogeneous in CB mode or the saw-tooth-like rough surface in W mode, and clarified that the wetting on the rough surface can involve multiple local free energy minima with different contact angles and that the contact angle of the global free energy minimum approaches to that of the conventional theory only when the scale of the liquid droplet is much bigger than the scale of the periodicity of the rough surface. Shahraz *et al*.^22^ computed the wetting free energies of every possible pinning points on the surface with periodic rectangular protrusions in W or CB mode, to predict the contact angles of the liquid droplet with finite volume at multiple pinning points. However, since the free energy landscape between the pinning points were not investigated, it cannot be guaranteed that all the pinning points investigated in the study are at local free energy minima.

In this work, we present a more generalized wetting theory by considering the entire free energy landscape including the transition configuration between the pinning points in three wetting modes, P, W, and CB, on a surface with rectangular protrusions as well as non-uniformly rough surfaces. First, we confirm that the pinning phenomena can be understood by the existence of multiple local minima of the free energy landscape separated by energy barriers. For all wetting modes (P, W, and CB), we show that the contact angle at the global minimum recovers the prediction of conventional wetting theory within 2° when the droplet size (diameter of the initial droplet) becomes at least 40 times larger than the characteristic scale of the surface roughness (periodicity of the texture). Second, we compute the free energy barriers between available pinning points in all wetting modes. For P and CB modes, the energy barrier between local minima tends to decrease as the contact angle becomes further apart from the contact angle at the global minimum energy and eventually becomes zero, which enables us to predict the ultimate bound of advancing and receding angles. Our theory is then applied to explain the measured contact angle on the surface with anisotropic roughness^19^. Finally, we calculate the free energy of the wetting on the non-uniformly rough surface and reaffirm in terms of free energy that the contact angle is determined by the roughness of the substrate near the three-phase contact line^20^,^23^, not by the overall roughness within the droplet-substrate contact area. When the effect of gravity is ignored^22^, the proposed theory is universally valid for any scale of droplets unless the droplet diameter is a few nanometers or smaller because we assume that the interfacial energy of rough surface can be written as the flat surface interfacial energy multiplied the ratio of true area to the apparent area. The length scale of the molecular interaction is known to be about one to two nanometers^24^. Our theory reproduces the conventional wetting theory in the limit of an infinite ratio between the droplet size and characteristic scale of surface roughness.

For the mathematical simplicity, we consider a two-dimensional (2D) finite-sized liquid droplet. As illustrated in Fig. 1a, we model the rough surface as periodic rectangular textures and model the boundary of the liquid droplet as an arc of a circle while ignoring the effect of gravity. In other words, our 2D droplet is a simplification of an infinitely long cylindrical droplet. Even though our theory cannot provide the exact quantitative prediction of contact angle of realistic spherical-cap-like 3D droplets, it can still offer qualitative physical understanding about the wetting of small droplet on rough surface, such as the origin of pinning, the wetting on non-uniformly rough surface, and contact angle change upon droplet volume change. The transition between the pinning points in CB and P modes is modelled by assuming that the three-phase contact line horizontally slides between the top of grooves. In W mode, we assume that the liquid tip forms a vertical straight line when inside a groove. While such an assumption may cause a small numerical difference, the resulting free energy landscape would correctly capture the multiple local minima and the energy barriers between them qualitatively well (See Supplementary Information).

We find that the free energy has local minima when one end of the droplet is fixed at either the left or right corner of the step. Two cases are illustrated in the inset of Fig. 1b. Because a hydrophobic surface prefers to reduce the contact area between the droplet and the substrate at a given contact angle, Case I has the lower free energy for the hydrophobic surfaces (θ_e_ > 90°), and Case II has the lower free energy for the hydrophilic surfaces (θ_e_ < 90°) (see Supplementary Information for more details). θ_e_ refers to the equilibrium contact angle on a flat surface determined by Young’s equation^1^, σ_LV_ cos θ_e_ = σ_SV_ − σ_SL_, where σ_LV_, σ_SV_ and σ_SL_ denote the liquid-vapor, solid-vapor and solid-liquid interfacial energies, respectively. For the two cases, the number of grooves below the liquid droplet n can be expressed with length of the baseline L, width of the step W, and width of the groove G, as described below.


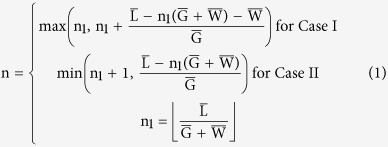


n_1_ is the number of groove-step pairs which fully covered with liquid, hence a natural number, and n is defined to account for partially filled grooves (real number). The overbar indicates the dimensionless variables. In what follows, all length and energy variables are normalized against the radius of the circular droplet R_0_ (i.e. the volume of droplet is 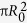) and σ_LV_R_0_, respectively. The values of n are visualized in Fig. 1b. for the case when 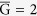 and 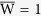. As illustrated in the figure, n continuously increases when the three-phase contact line is on the groove and is conserved when the contact line is on the step, as 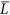 increases. Thereafter, the radius of the curvature and the free energy of each wetting modes can be derived in terms of n, geometrical factors, and θ, which are presented in Table 1. Details on the numerical calculations are described in the Supporting Information. Because the current study considers only 2D droplet cases, the effect of the line tension^25^ can be ignored. However, one needs to incorporate the line tension effect when dealing with 3D droplets on textured surfaces^26^,^27^.

Based on the free energy expression as a function of θ, we can find the allowable contact angles with free energy minima at a given roughness factor^28^ 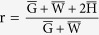, i.e., the ratio of the true surface area to the projected area. For example, Fig. 1c–e show the relationship between the free energy and the contact angle for the W mode when r = 1.2, 1.5 and 1.8, respectively when 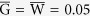 (i.e., a droplet size of 2R_0_ is 20 times that of the characteristic scale of the surface roughness, G + W). The vertical dotted line is the contact angle determined by the conventional wetting theory on the Wenzel mode, cos θ = r cos θ_e_. The contact angles at the global free energy minima under different surface roughnesses, which are highlighted by the blue circle, green triangle, and red square, do not match perfectly with the prediction from the conventional wetting theory. Thereafter, one can predict the contact angle as a function of the surface roughness factor r by connecting the contact angles at the global free energy minima, as illustrated in Fig. 1f. The conventional wetting theory prediction (red dotted curve), cos θ = r cos θ_e_, is also presented in comparison. The contact angles for the CB and P modes can be obtained in a similar way as functions of 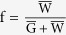, which is the fraction of the step area from the projected area.

We then consider how the contact angle changes with the droplet size by varying the dimensionless variable 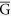, which is the ratio of the groove width to the initial droplet radius. For a given 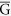, the roughness factor^28^ r and the step fraction f can be tuned by changing the height or width of the steps, 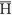 or 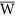. The predicted contact angles for W, CB, and P modes are presented in Fig. 2a–d with varying values of r and f. When 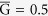, i.e., the droplet size is 2 times the characteristic scale of the surface roughness, our theory and the conventional theory^10^,^11^,^12^ predict different contact angles because the wetting free energy landscape of a small droplet is extremely different from that of a large droplet. Hence, the conventional theory should not be used. However, in case of 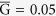, the contact angle from our theory converges to that from the conventional theory within the range of ±10°. When 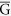 reaches 0.005, the contact angle from our theory recovers the prediction of conventional theory^10^,^11^,^12^ almost perfectly, which is expected. Considering the typical resolution of the contact angle measurements (1~2°)^15^, we suggest the conventional theory^10^,^11^,^12^ should be applied in the case when 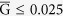 (see Supplementary Information for details), i.e., when the droplet size is at least about 40 times bigger than the characteristic scale of the surface roughness.

We can predict the most stable wetting mode for the substrates with different Young’s angle θ_e_ by comparing the free energies of the three modes^29^. As depicted in Fig. 3d, we find that in accordance with the conventional theory, on a hydrophilic surface (θ_e_ < 90°), the contact angle is given by the higher contact angle between the predictions based on the P mode and W mode and on the hydrophobic surface (θ_e_ < 90°) by the lower contact angle between the predictions based on the CB mode and W mode. The choice between the W mode and the CB mode is made by comparing the free energy values in Table 1. To select between the W mode and the P mode, we use the critical contact angle theory^2^ instead of the direct free energy comparison because the initial free energy of the P mode differs from the other modes. The critical contact angle, θ_C_ is determined when the free energy variation to fill an additional groove is 0. If the contact angle of the flat surface θ_e_ is smaller than θ_C_, the free energy variation to fill another groove becomes negative, spreading occurs, and the Penetrate mode is selected. In our work, the critical contact angle can be expressed with geometric factors as 
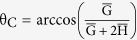
. As illustrated in Fig. 2d, for the case of 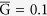, r = 1.5, and f = 0.5, one can find that the most stable wetting mode curve follows a similar path as that of the conventional theory.

We then confirm the origin of the pinning effect is the occurrence of multiple local minima and calculate the free energy barriers in terms of geometric factors for the three wetting modes. In the W mode, a drastic free energy change occurs when the three-phase contact line is located near the corner of the step because the additional boundary may form or disappear. The A’ and B’ points at the end of the step in Fig. 3a include an additional boundary line while A and B do not. As depicted in Fig. 3a, the free energy differences between A’ and A(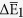) or B’ and B(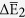) act as the primary free energy barrier and form a local free energy minimum. The amount of the energy barrier can be expressed with 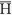 and θ, as shown in Table 2. When the substrate is hydrophobic, the local minimum point is located on B, and one can notice that this corresponds to the experimentally observed three-phase contact line location^19^ when pinning occurs. A similar discussion can be repeated for the Cassie-Baxter or the Penetrate mode. By calculating the free energy of each point of Fig. 3b and c, the energy barriers can be formulated, as summarized in Table 2. The superscripts A and B refer to the points A and B in the figures, respectively, and the subscripts C and P refer to the wetting modes. Interestingly, while the Wenzel mode always has local free energy minima because of the additional liquid-vapor boundary line, the Cassie-Baxter or the Penetrate mode do not possess local minimum points when θ < θ_e_ or θ > θ_e_, respectively. The disappearance of the energy barrier indicates the ultimate bound of advancing and receding angles which are determined by available local free energy minima. The maximum advancing (receding) angle can be obtained from experiments will be the highest (lowest) contact angle at the pinning points with nonzero energy barrier. We speculate that the conventional Wenzel state has very large contact angle hysteresis because of the non-vanishing energy barrier between local minima^30^.

Now, we apply the proposed theory to understand the wetting experiments on the surface with anisotropic roughness^19^. An experiment performed by Chen *et al*.^19^ used morphologically patterned surface to measure the contact angle of the surface with anisotropic roughness made of PDMS (θ_e_ = 114°) along both perpendicular and parallel directions. The width of step and groove were 23 μm and 25.6 μm each. The height of the step was 30 μm. The measurement of the contact angle were conducted with water droplet of volume from 0.59 mm^3^ to 5.679 mm^3^. They confirmed that droplets are in the CB mode, and measure the number of pillars filled by or the base line length of the droplet. It was reported that the contact angle at the direction perpendicular to the grooves is likely to be similar to the advancing contact angle^31^,^32^, and that the advancing contact angle is chosen as the maximum contact angle among the local free energy minima^33^. However, since there exists an external energy perturbation such as ambient vibration and the inertia associated with the droplet spreading, we expect that the measured advancing contact angle must be smaller than the maximum contact angle among local minima. We compute the energy barrier at each local minima for different droplet volumes, and compared the energy barrier at the measured contact angle for corresponding droplet volume. We could see that the free energy barrier between B state and C state in Fig. 3b plays a main role in determining the advancing contact angle in CB mode. The energy barrier can be calculated by the formula in Table 2. Since the energy barrier formula is non-dimensional, we multiply R_0_σ_LV_ and the baseline length parallel to the groove direction to dimensionalize the formula to compare the energy barriers for different droplet volume. The energy barrier between B state and C state, at each local minimum is plotted in Fig. 4a. Each dot in the curves refers to the energy barrier at each pinning point and all open symbols refer to the experimental result for different droplet volume. In the figure, one can notice that the energy barriers for different droplet volumes are similar. The average energy barrier is represented with the black dotted horizontal line which is located close to all open symbols. One may consider the black dotted line as the typical external energy perturbation in the series of experiments reported in the study. The contact angle for specific liquid volume can be predicted by capturing the pinning point which has the smallest distance from the average energy barrier (black dotted in Fig. 4b). The predicted contact angle agrees well with experimental results and show the same trend with experiments. One may perform a similar analysis to predict the receding contact angle or the contact angle hysteresis of other experiments.

We then apply our theory to analyse a surface with non-uniform roughness that has a rough center and flat periphery. In other words, we compute the free energy landscape when there exists an upper bound n_MAX_ in the number of filled grooves, n. For example, we compute the free energies of the wetting states when 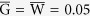 and θ_e_ = 120° as functions of contact angle θ with n_MAX_ = 14 and 19, as depicted in Fig. 5a and b, respectively. Figure 5a illustrates the situation where the area of the rough central region is small enough that the three-phase contact line is located on the flat region. We find that the global free energy minimum is located at the Young’s angle θ_e_. It is the case for any n_MAX_ ≤ 14 because the free energy curve with a fixed n has a minimum at θ_e_, as depicted by the red dotted curves in Fig. 5a. On the contrary, Fig. 5b shows a case where the area of the rough region is large enough and the contact line is located within the rough center. In this case, the local minimum associated with n = n_MAX_ = 19 has a higher free energy than the global minimum. Hence, the contact angle prediction becomes identical to the surface with uniform roughness. Our results agree with the previously proposed wetting theory for a non-uniform rough surface^20^,^23^, which proposed that the contact angle is determined by the roughness condition near the three-phase contact line. Our work offers an extended theory that enables us to predict whether the contact line will be located on the rough center or on the flat region. We note that our theory can be generalized to study the wetting on surfaces with a more complex non-uniform roughness or be used to build a surface to fix the droplet to the specific location^34^, complementing the pioneering works by Johnson and Dettre^35^,^36^.

In conclusion, we have developed a wetting theory that can predict the wetting angle and wetting mode when the liquid droplet is not much larger than the surface texture scale. Because the conventional theory assumes a much larger size of the droplet compared to the texture scale, there have been limitations in how to analyse experiments that investigate the wetting of small liquid drops. Our theory suggests that conventional theory should be used when the droplet size is about 40 times larger than the characteristic scale of the surface roughness and provides a deeper physical understanding regarding the wetting of smaller liquid droplets on non-uniform rough surfaces.

## Additional Information

**How to cite this article**: Kim, D. *et al*. Wetting theory for small droplets on textured solid surfaces. *Sci. Rep*. **6**, 37813; doi: 10.1038/srep37813 (2016).

**Publisher's note:** Springer Nature remains neutral with regard to jurisdictional claims in published maps and institutional affiliations.

## Supplementary Material

Supplementary Information

## Figures and Tables

**Figure 1:**
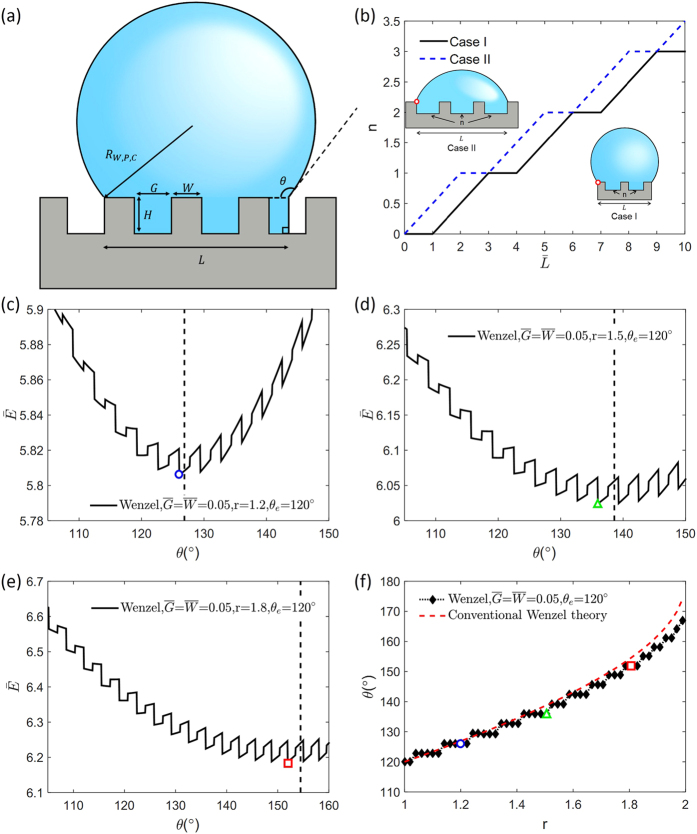
Assumptions and methods to achieve r − θ relation. (**a**) Schematic of liquid droplet and the 2D textured solid substrate. To simplify the problem, we assume that the liquid droplet has a vertical surface when the non-pinned tip is on the groove (**b**) Relation between n and 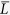 when 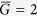, and 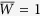 for two locally pinned states. (**c**–**e**) Relation between θ and 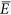. Global minimum of free energy can be found at various roughness factors r. (**f**) Relation between r and θ. The contour is constructed by contact angles of the global free energy minima at each roughness factor r.

**Figure 2:**
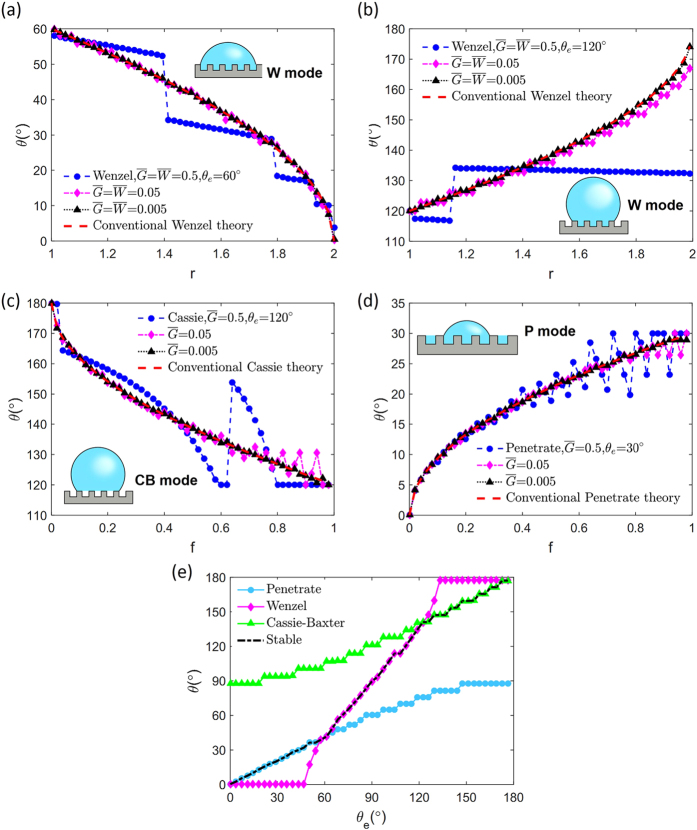
Results of contact angle and wetting mode prediction from the proposed theory. (**a**–**d**) Convergence curve of each wetting modes ((**a**) Wenzel (hydrophilic), (**b**) Wenzel (hydrophobic) (**c**) Cassie-Baxter, (**d**) Penetrate). Conventional wetting theory should be used when 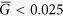 (**e**) Wetting mode selection with respect to θ_e_ when 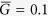, r = 1.5 and f = 0.5.

**Figure 3:**
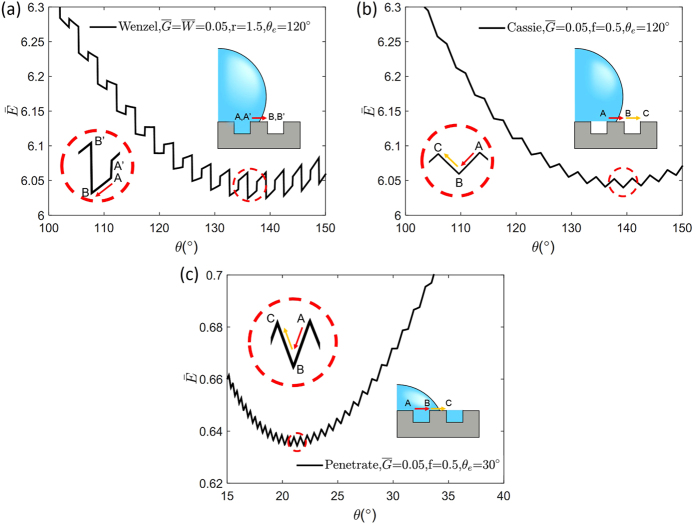
Relation between θ and 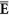 with respect to wetting modes. (**a**) Wenzel, (**b**) Cassie-Baxter, (**c**) Penetrate. Equations of dimensionless energy barriers are presented in Table 2.

**Figure 4:**
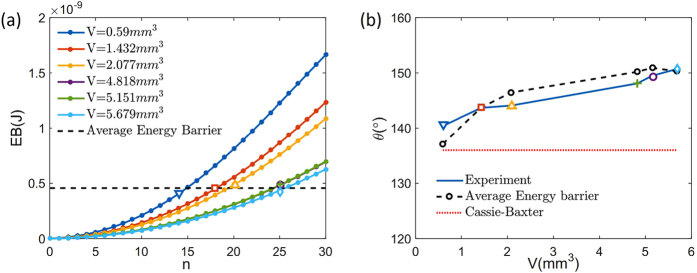
Energy barrier and experimental results on surface with anisotropic roughness. (**a**) Amount of energy barrier at the pinning point of surface with anisotropic roughness. The experimental data are signed with symbols (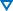, 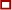, 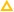, 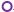, 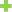, 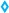) and one can notice that they have similar energy barriers. (**b**) Predicted contact angle with average energy barrier. The predicted contact angle shows good match with experimental results.

**Figure 5:**
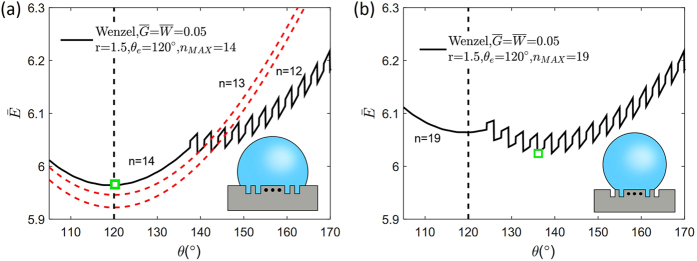
Relation between θ and 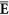 of the Wenzel mode when the surface has non-uniform roughness. The contour contains slices of free energy contours of fixed n (red dotted). The global free energy point is highlighted with the green square symbol (**a**) The global free energy minimum point might be located on the n = n_MAX_ curve or (**b**) the global free energy point of the surface with uniform roughness.

**Table 1:** Expressions of curvature radius and free energies of three wetting modes.

	Curvature Radius 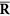	Free Energy 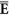
W	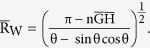	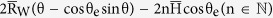
		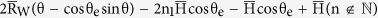
CB	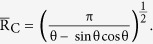	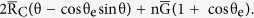
P	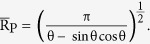	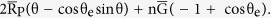

Overbar indicates dimensionless variables.

**Table 2:** Dimensionless energy barriers for each wetting mode.

Wetting Mode	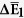	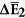
Wenzel	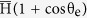	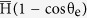
Cassie-Baxter	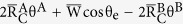	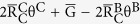
Penetrate