# Supplementary material for: Wetting theory for small droplets on textured solid surfaces
Source: Sci Rep. 2016 Nov 29;6:37813. doi: 10.1038/srep37813 (PMC5126673; doi:10.1038/srep37813)
Supplement: Supplementary Information [file srep37813-s1.pdf]

Supplementary Information

Wetting theory for small droplets on textured surfaces

Donggyu Kim[1], Nicola M. Pugno[2], and Seunghwa Ryu[*,1]

Affiliations

[1] Department of Mechanical Engineering, Korea Advanced Institute of Science and Technology (KAIST), 291 Daehak-ro, Yuseong-gu, Daejeon 305-701, Republic of Korea

[2] Department of Civil, Environmental, and Mechanical Engineering, University of Trento, Trento, Italy

[*]Corresponding author email : ryush@kaist.ac.kr

Supplementary Figure 1: Free energy comparison between circular boundary and vertical boundary

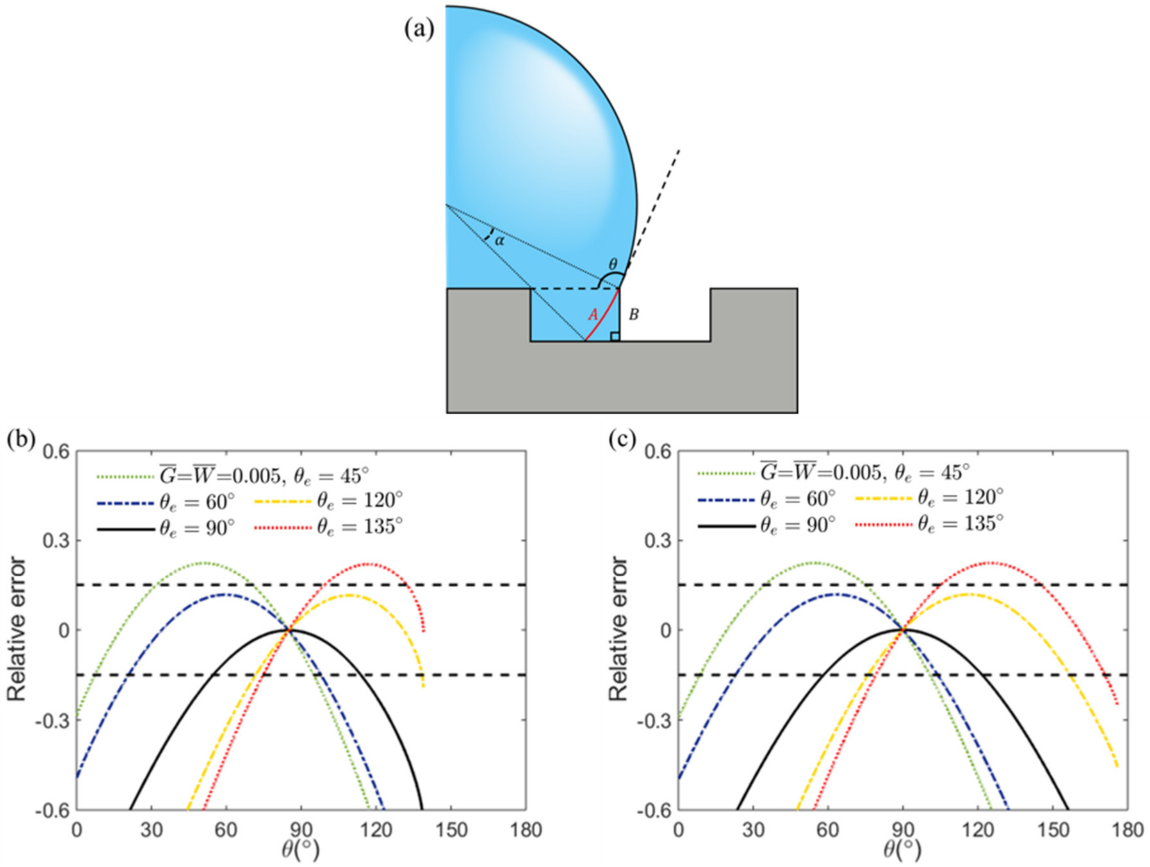

Supplementary Figure S1. Free energy comparison between circular boundary and vertical boundary. (a) Schematic of circular (A) and vertical (B) boundary. (b) Relative error between free energy of two boundary assumptions. The surface was set by $\bar{G} = \bar{W} = 0.5$, r=1.5. (c) Relative error between free energy of two boundary assumptions. The surface was set by $\bar{G} = \bar{W} = 0.005$, r=1.5. Rather than the droplet volume, the Young's angle mainly affects the error arising from the vertical boundary assumption.

Supplementary Figure 2: Free energy curve of a droplet in Wenzel mode

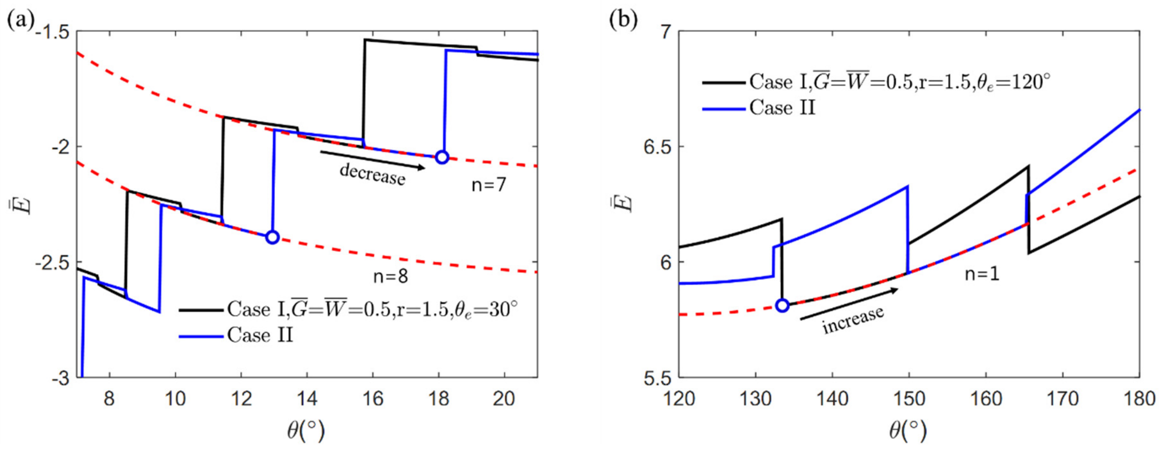

Supplementary Figure S2. Convex curvature of Case II is always on the right side of Case I. Because $\bar{\mathbf{E}}$ decreases for (a) a hydrophilic substrate and increases for (b) a hydrophobic surfaces as $\boldsymbol{\theta}$ increases, the former has a minimum at Case II and the latter has a minimum at Case I.

Supplementary Figure 3: Convergence to conventional theory with respect to $\bar{G}$ for each wetting mode

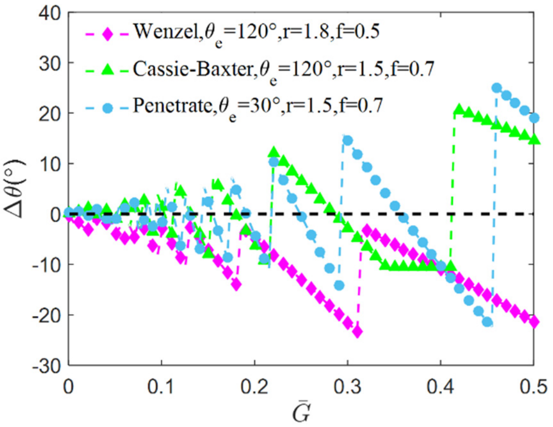

Supplementary Figure S3. Convergence to conventional theory due to $\bar{G}$ for each wetting mode. $\bar{W}$ *and* $\bar{H}$ are set to satisfy the roughness factors (r, f) in the legend. The contact angle difference between the proposed theory and the conventional theory ($\Delta\theta$) converges to $1\sim2°$ when $\bar{G} \le 0.025$

Supplementary Note 1: Free energy comparison between circular boundary and vertical boundary

It was reported that the liquid droplet forms the part of the circle when the effect of the gravity is neglected[1]. However, it is mathematically very hard to model the liquid droplet on periodic rectangular protrusion with circular boundary with the constant droplet volume constraint. Therefore, we used vertical liquid boundary (path B in **Fig. S1a**) to model the droplet in Wenzel mode (CB mode and P mode do not need the assumption) rather than the circular liquid boundary (path A in **Fig. S1a**). To check the validity of our assumption, we compare the free energy of two boundary for a simple case where the three-phase contact line touches the bottom of the groove.

The free energy of each liquid boundary can be formulated as below.

$$\bar{E}_C = \bar{R}_W \alpha$$

$$\bar{E}_V = \bar{H} + (\bar{R}_W \sin(\theta + \alpha) - \bar{R}_W \sin(\theta)) cos\theta_e$$

Here, $\bar{E}_C$ and $\bar{E}_V$ refer to the free energy of the circular and the vertical boundary. The free energy can be formulated by $\mathrm{E} = \sigma_{\mathrm{LV}} A_{LV} + (\sigma_{SL} - \sigma_{SV}) A_{SL}$. α refers to the angle between circular boundary within the groove as depicted in **Fig. S1a**. The relative error between two energy barriers can be defined by $\frac{\bar{E}_C - \bar{E}_V}{\bar{E}_V}$. The relative error between two boundaries are calculated for the surface with r=1.5, when $\bar{G} = \bar{W} = 0.5$ (small droplet) and $\bar{G} = \bar{W} = 0.005$ (large droplet). The region with high contact angle ($\theta > 140°$) when the droplet volume is small is not investigated because α cannot be decided in the case because the three-phase contact line cannot reach the floor of the groove. As shown in **Fig. S1b and Fig. S1c**, the relative error is smaller than 15% unless the surface is highly hydrophilic ($\theta_e < 60°$) or highly hydrophobic ($\theta_e > 120°$). Because the droplet on the highly hydrophilic (hydrophobic) surface prefers P (CB) mode, we can conclude that our assumption is reasonable within 15% of errors.

Supplementary Note 2: The choice between Case I and Case II along wetting modes

1-1. Cassie-Baxter & Penetrate mode

From Young's relation[2], one can say the free energy from same area of liquid - solid boundary is always larger than that from the liquid – vapor boundary for any substrate when $\theta > 0°$. Because Case II always contains larger n than in Case I, regardless of L (**Fig. 2b**), Case I contains less area of the liquid-vapor boundary. Consequently, Case I become more stable if the same θ is assumed. In the case of Penetrate mode, on the contrary, Case II become more stable because the liquid-vapor boundary is substituted by a liquid-liquid boundary, which contains 0 surface energy.

1-2. Wenzel mode

If the same amount of n is assumed, Case I always has a larger $\bar{L}$ or smaller θ than Case II. As we noted in the text, the $\bar{E} - \theta$ graph contains convex contours of conserved n, where the minimum is at θ_e (red dotted in **Figs. S1a**, **S1b**). Because Case I has a larger θ for conserved n, one can notice the convex contour of Case II is always on the right side of Case I for conserved n. Recalling that the Wenzel mode has a larger equilibrium contact angle than θ_e, when the substrate is hydrophilic, the free energy of the liquid decreases as θ increases. Therefore, the local free energy minimum near the equilibrium contact angle is on the right side of the contour, Case II. If a hydrophobic substrate is assumed, by the opposite logic, the local free energy minimum is on Case I.

Supplementary Note 3: Convergence to conventional theory with respect to $\bar{G}$ for each wetting modes

As depicted in **Figs. 2a-c**, the predicted contact angle from the proposed theory (θ_P) converges to the contact angle from the conventional theory (θ_T) when $\bar{G} \leq 0.025$. We are not able to find a closed form expression for the difference between the two contact angles ($\Delta\theta = \theta_P - \theta_T$), but find that the envelope of the oscillating $\Delta\theta$ curve decreases with $\bar{G}$ for all wetting modes (**Fig. S2**). The contact angle difference converges with vibration and becomes $1 \sim 2°$ range when $\bar{G} \leq 0.025$.

Supplementary Note 4: Free energy and curvature radius calculation about θ

The curvature radius of the liquid droplet of each wetting mode (W,CB,P) can be formulated with a circular trace of the boundary of the liquid and the constant volume constraint as Shahraz et al. [1] reported. In Wenzel mode, the sum of the area of the circular part and of the groove should be constrained. Therefore, the curvature radius of the Wenzel mode $\overline{R}_W$ can be formulated as follows.

$$\overline{R}_W = \left(\frac{\pi - n\overline{G}\overline{H}}{\theta - \sin\theta\cos\theta}\right)^{\frac{1}{2}}$$

After the curvature radius is formulated, the free energy of the droplet can be calculated by summing up the free energy from the liquid-vapor boundary and the liquid-solid boundary as follows.

$$E = \sigma_{LV}A_{LV} + (\sigma_{SL} - \sigma_{SV})A_{SL}$$

A_{LV} or A_{SL} refer to the area of the boundary between the liquid and the vapor or solid and the liquid. Employing the curvature radius formula, the free energy of the droplet in Wenzel mode can be shown.

$$\overline{E}_W = 2\overline{R}_W(\theta - \cos\theta_e\sin\theta) - 2n\overline{H}\cos\theta_e \ \ (n \in \mathbb{N})$$

$$\overline{E}_W = 2\overline{R}_W(\theta - \cos\theta_e\sin\theta) - 2n_1\overline{H}\cos\theta_e - \overline{H}\cos\theta_e + \overline{H} \ (n \notin \mathbb{N})$$

Because an additional boundary of the liquid-vapor boundary exists when $n \notin \mathbb{N}$ in Wenzel mode, the free energy expression either differs when $n \in \mathbb{N}$ or not. Similar analysis can be repeated for the Cassie-Baxter mode and Penetrate mode to include the curvature radius or free energy of the liquid in CB mode or P mode. In particular, in the case of P mode, we only model the liquid forming the droplet to construct R_0 and set the initial state of the free energy with

a rough surface in which grooves are filled with liquid for convenience. Then the free energy and the curvature radius of the penetrate mode can be formulated similar with CB mode as follows.

$$\overline{R}_{C,P} = \left(\frac{\pi}{\theta - \sin\theta\cos\theta}\right)^{\frac{1}{2}}$$

$$\overline{E}_{C,P} = \overline{R}_{C,P}(\theta - \cos\theta_e \sin\theta) + n\overline{G}(1 + \cos\theta_e)$$

Because the $n - \theta$ relation is known if the liquid tip condition (Case I or Case II) is suggested, a set of $(\overline{R}, n, \overline{E})$ can be shown with respect to a specific θ. In the case of CB mode and P mode, because $\overline{R}_{C,P}, \overline{E}_{C,P}$, and n are explicitly expressed by θ, a pair of $(\overline{R}_{C,P}, n, \overline{E}_{C,P})$ can be easily obtained for a specific θ. In W mode, $\overline{R}_W, \overline{E}_W$, and n are in an implicit relation; therefore we adopted the bisection method to numerically calculate the set of $(\overline{R}_W, n, \overline{E}_W)$. With this process, the free energy of the droplet can be numerically obtained from a specific θ and can be used to find the contact angle of the minimum free energy.

References

1 Shahraz, A., Borhan, A. & Fichthorn, K. A. A Theory for the Morphological Dependence of Wetting on a Physically Patterned Solid Surface. *Langmuir : the ACS journal of surfaces and colloids* **28**, 14227-14237, (2012).

2 Young, T. An Essay on the Cohesion of Fluids. *Philosophical Transactions of the Royal Society of London* **95**, 65-87, (1805).

3 Chen, Y., He, B., Lee, J. H. & Patankar, N. A. Anisotropy in the wetting of rough surfaces. *J Colloid Interf Sci* **281**, 458-464, (2005).
